# Letter to the editor UC-II® Undenatured type II collagen: update to analytical methods

**DOI:** 10.1186/s12970-019-0298-3

**Published:** 2019-07-16

**Authors:** James P. Lugo

**Affiliations:** Lonza Inc., 5451 Industrial Way, Benicia, CA 94510 USA

**Keywords:** Undenatured type II collagen, ELISA assay, Total collagen assay

## Abstract

Supplementation with UC-II® undenatured type II collagen has been shown to provide joint health benefits for both healthy adults and adults with knee osteoarthritis in controlled clinical trials. These trials used UC-II® materials with undenatured type II collagen characterized by an ELISA method that utilizes a monoclonal antibody specific for epitopes expressed by undenatured type II collagen protein only. In 2014, we modified the sample preparation part of the ELISA method in order to reduce the amount of time devoted to this procedure. We undertook these modifications in order to provide commercial manufacturers with a streamlined assay methodology better aligned with their product testing requirements. In doing so, it altered the percent of undenatured collagen now reported for UC-II® undenatured type II collagen. The intent of this letter is to clarify that the UC-II® materials used in the published clinical research cited herein, and the commercially available ingredient, remain identical and to describe the rationale for the change in the extraction method.

To the Editor:

In 2013, my colleagues and I demonstrated that healthy adults supplemented with UC-II® undenatured type II collagen presented with improved post-exercise knee extension and comfort [1]. In 2016, we described similar benefits for joint comfort, flexibility, and physical function in adults with knee osteoarthritis [2]. The investigational materials used in both studies derived from commercial batches characterized using a sensitive enzyme-linked immunosorbent assay (ELISA) method adapted from published procedures [3].

In between the publication of these two trials, we updated our extraction method in order to reduce the overall time required for quantifying the amount of undenatured collagen type II. While this new method offers time saving advantages, due to the shorter extraction time, the new assay may indicate lower collagen type II levels. However, the method is sufficient to confirm the minimum specification requirements for this ingredient. As a result, the different assay methods yield differing amounts of undenatured type II collagen for the same material tested (Table 1).

**Table 1:** Comparison of Assay Methods Characterizing UC-II® Undenatured Type II Collagen

UC-II® Study Design	Research Material (Lot No.)	Undenatured Type II Collagen (in 40-mg UC-II® material)	
		Per ELISA-Old Assay	Per ELISA-New Assay
Efficacy study - healthy adults*	1109006	10.8 mg (27.0%)	2.3 mg (5.8%)
Efficacy study – people with osteoarthritis **	1204004	10.8 mg (27.1%)	1.2 mg (3.0%)
Typical lot sold into commerce	1309029	10.6 mg (26.5%)	1.3 mg (3.3%)

ELISA indicates enzyme-linked immunosorbent assay

*Lugo et al. J Int Soc Sports Nutr. 2013;10:48

**Lugo et al. Nutr J. 2016;15:14

Despite the change in the extraction protocol, it is important to note that the UC-II® undenatured type II collagen material itself has not changed. The material used in these clinical studies is produced using the same manufacturing processes that have been in place over the past 14 years.

The total collagen assay, developed under Good Laboratory Practices and validated per ICH guidelines, offers key advantages over the previously used ELISA method. Overall, the new assay is a faster, more straightforward procedure that is easily transferable to other laboratories.

In summary, the UC-II® material used in the published clinical research and the commercially available ingredient remains identical. The only change is to the extraction method used to prepare the UC-II® undenatured type II collagen material prior to ascertaining its percentage of undenatured type II collagen.

## Data Availability

Not applicable

## Abbreviation

ELISA: Enzyme-linked immunosorbent assay
